# Impact of treatment delay of multiple myeloma bone disease on later myeloma-related skeletal events and outcome^☆^

**DOI:** 10.1016/j.jbo.2025.100693

**Published:** 2025-06-07

**Authors:** Eskelinen Veera, Niemi Pauli, Partanen Anu, Kangas Jaakko, E.L.Kuusisto Milla

**Affiliations:** a Department of Medicine, University of Oulu, Oulu, Finland; b Department of Medicine, Kuopio University Hospital, Kuopio, Finland; c Hematology-Oncology Unit, Oulu University Hospital, Oulu, Finland; d Biomedicine and Internal Medicine Research Unit, University of Oulu, Oulu, Finland; e Medicine Research Unit, Oulu University Hospital, Oulu, Finland; f Department of Internal Medicine, Länsi-Pohja Central Hospital, Kemi, Finland

**Keywords:** Multiple myeloma, Bone disease, Treatment delay, Fracture

## Abstract

Limited data exists on the effect of treatment delay of multiple myeloma (MM) bone disease on the disease course. In this real-world analysis of 625 patients with newly diagnosed MM (NDMM) we aimed to investigate the impact of delay in starting bone disease treatment on later skeletal events and outcome.

Altogether 480 (76.8 %) patients had bone disease at the diagnosis, 282 (45.1 %) patients had a fracture at diagnosis, and 181 (29.1 %) patients had a later fracture during the follow-up. A delay in the initiation of treatment of bone disease was experienced by 221 (35.4 %) patients and tooth extraction was a main reason for the delay. Patients with a delay seemed to experience earlier and more frequent later fractures. Also, a fracture (p = 0.003) or bone disease (p < 0.001) at diagnosis predicted earlier incidence of later fractures. As a bone targeted treatment, altogether 363 (58.1 %) patients received zoledronic acid, 81 (13.0 %) denosumab and 134 (21.4 %) other bone-targeted treatment. Patients treated with denosumab had poorer overall survival (OS) (p < 0.001) and experienced earlier later fractures (p = 0.003). Multivariate analysis showed that bone disease at diagnosis (p = 0.043) and given bone disease treatment (p = 0.023) significantly impacted on the time to next fracture. Regarding OS, delay in osteoprotective treatment (p = 0.004) and time of the diagnosis (p < 0.001) were significant factors in multivariate analysis.

To conclude, this study suggests that early initiation of bone disease treatment seemed to prevent later fractures. These findings highlight the importance of patients’ rapid access to a dentist and the start of bone targeted treatment without delay after a myeloma diagnosis.

## Highlights

• A real-world data with detailed information on MM patients’ dental care.
• The main reason for a delay was tooth extraction.
• Tooth extraction at diagnosis stage increases the risk for ONJ.
• A treatment delay in bone disease may predict earlier and frequent later fractures.
• Fracture and bone disease at diagnosis predict earlier later fractures.

## Graphical abstract

**Figure:**
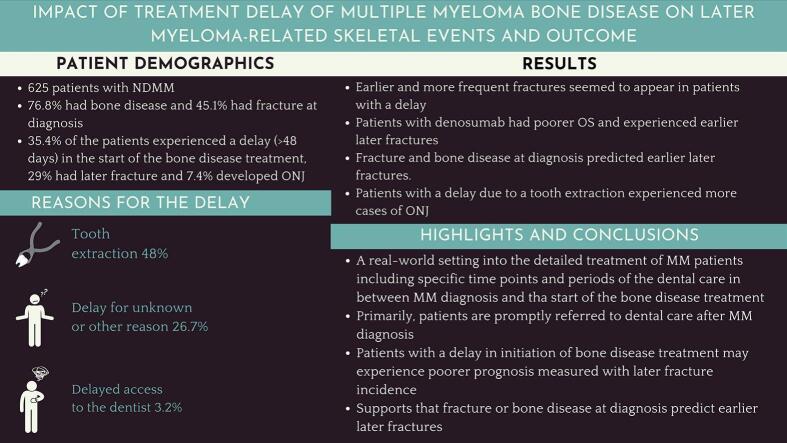

## 1 Introduction

Multiple myeloma (MM) is a malignancy of clonal B-cell derived plasma cells. A great majority of the patients have a typical myeloma-related osteolytic bone disease already at diagnosis due to increased osteoclast activity and diminished osteoblastogenesis caused by interactions between bone microenvironment and pathological myeloma cells [1]. Body weight, the need for central nervous system affecting pain medication and high pain score have been proposed to be predictive factors for the bone disease combined with related morbidities such as fractures or compressions with need for immediate actions [2]. Most remarkable, a large Swedish real-world registry study observed excess mortality in patients with bone fractures both at diagnosis and especially in those with later fractures after diagnosis [3] in concordance with the findings by Terpos et al [2]. Accumulated data on the detailed mechanism on the pathology of bone disease have been crucial for the development of targeted drugs like bisphosphonates and denosumab [4].

The oral cavity is a potential source of bacteremia, infections, and related morbidities in cancer patients highlighting the importance of oral health examination at diagnosis [5]. The International Myeloma Working Group (IMWG) guidelines recommend treating dental disturbances before starting bone disease treatment [6] to prevent infections and osteonecrosis of the jaw [7]. Dental surgery or invasive procedures should be avoided during bisphosphonate treatment or bisphosphonate should be paused for three months before and after operations [6]. However, in clinical practice shorter pauses are sometimes used to avoid delaying bone disease treatment.

A previous retrospective study concluded that the start of bisphosphonates within two months after the diagnosis of symptomatic MM is associated with a prolonged time to appearance of skeletal-related events (SREs) [8]. Limited data exist on the reasons of delay in the treatment of the myeloma bone disease and the impact of the delay on the occurrence of later myeloma SREs.

In the present real-word registry study, we aimed to investigate the delay of the start of treatment against myeloma bone disease, reasons for delay as well as the effect of delay on outcome and emergence of new skeletal-related phenomenon.

## 2 Patients and methods

**Fig. 1:**
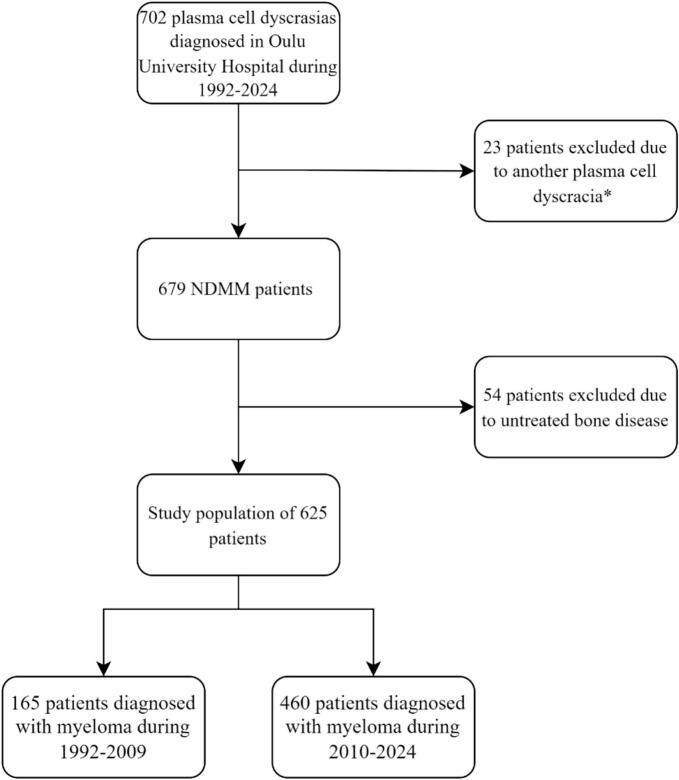
Flow chart of the study population of patients with newly diagnosed myeloma (NDMM). *Monoclonal gammopathy of undetermined significance (MGUS), Smouldering multiple myeloma (SMM), amyloidosis, Monoclonal gammopathy of renal significance (MGRS).

The study population consisted of 625 patients with NDMM who were treated in Oulu University Hospital during the years 1992–2024. See Fig. 1. Patients were divided into two groups based on their year of diagnosis (1992–2009 vs. 2010–2024), as treatment protocols and diagnostics, including imaging methods, have evolved significantly over this long period (1992–2024). Altogether 339 (54.2 %) of the patients included were men and 286 (45.8 %) were women. The median age at diagnosis was 65 years (range 25–90). Detailed patient characteristics are presented in Table 1.

**Table 1:** Characteristics of 625 newly diagnosed multiple myeloma patients.

Characteristics	n (%)
Gender (male)	339 (54.2)
Gender (female	286 (45.8)
Bone disease* at diagnosis	480 (76.8)
Imaging method at diagnosis	
X-ray	160 (25.6)
MRI	134 (21.4)
CT	247 (39.5)
NA	84 (13.4)
Fracture at diagnosis	282 (45.1)
Defined as pathological	200 (70.9)
Induction treatment	
PI-based	371 (59.4)
IMiD-based	79 (12.6)
Anti-CD38-based	8 (1.3)
Other^1^	147 (23.5)
Calcium plus Vitamin D supplementation before diagnosis	
Started before diagnosis	128 (20.5)
Started at diagnosis	428 (68.5)
Missing	112 (17.9)
Bone disease treatment	
Zoledronic acid	363 (58.1)
Denosumab	81 (13.0)
Other^2^	134 (21.4)
Later fracture	181 (29.0)
Pathological	116 (64.1)
Traumatic	30 (16.6)
Osteoporotic	29 (16.0)
NA	6 (3.3)
Removable teeth at diagnosis	140 (22.4)
Toothless at diagnosis	63 (10.1)
Osteonecrosis of the jaw developed	46 (7.4)
A delay in the start of bone disease treatment	221 (35.4)
No delay in the start of the bone disease treatment	224 (35.8)

IMiD immunomodulatory drug, NA not available, PI proteasome inhibitor. ^1^VAD vincristine-doxorubicin-dexamethasone, MP melphalan plus prednisone, CP cyclophosphamide plus prednisone, PAD bortezomib-doxorubicin-dexamethasone. Three patients did not receive antimyeloma treatment in induction but were included in the study because they had treatment for their myeloma (corticosteroids as well as bone disease treatment). ^2^pamidronate, alendronate, calcitonin. *Bone disease was defined according to IMWG guidelines.

At diagnosis, a total of 480 patients (76.8 %) had a myeloma-related bone disease. The imaging methods to detect bone disease are described in Table 1. Fracture was present in 282 (45.1 %) patients at diagnosis, and 200 (70.9 %) of these fractures were defined as pathologic and 82 (29.1 %) were osteoporotic or traumatic. The quality of the fracture was determined by an experienced clinical radiologist. As induction therapy for myeloma, proteasome inhibitor (PI)-based treatment was given to 371 (59.4 %), immunomodulatory drug (IMiD)-based to 79 (12.6 %), anti-CD38-based treatment to eight (1.3 %) and 144 (23.0 %) patients received other induction treatment. The median number of induction cycles given was four (range 1–21).

### 2.1 Statistics

Patient information was analyzed with IBM SPSS Statistics version 29.0.1.0 (171). Fisher's Exact Test and Chi-Square test were used for variable comparison. The ROC curve was used to assess the time value for a delay in bone disease treatment. Univariate analysis was conducted using ANOVA, and factors that showed statistical significance in the univariate analysis were further analyzed using Cox regression for multivariate analysis. Time to later fracture (TTLF) was calculated from the time of diagnosis date to next fracture for any reason or last follow-up date, whichever came first. Survival analysis was made with Kaplan-Meyer with log-rank test. Overall survival (OS) was defined as the time from diagnosis to last follow-up date or progression or death of any cause, whichever came first. Values of p < 0.05 were considered significant.

### 2.2 Ethics

This study was approved by the local ethical committee of Northern Ostrobothnia Hospital District (186/2020). The Declaration of Helsinki was followed in this study.

## 3 Results

Median follow-up time was 41 months (range 0–346). At diagnosis, fracture was diagnosed in 282 (45.1 %) patients and 181(29.1 %) patients had later fracture during the follow-up. For 89 (14.2 %) patients the last follow-up status was relapse or progression, whereas a total of 175 (28.0 %) patients had not yet experienced relapse. In total 359 (57.4 %) patients died during the follow-up and 313 (87.2 %) of those due to myeloma.

Univariate and multivariate analyses were performed on various factors that might have impacted TTLF or OS. Factors that had statistical significance are collected into Table 2. Bone disease at diagnosis and given bone disease treatment persisted significant in the multivariate analysis for TTLF. Instead, regarding OS, delay in starting of bone disease treatment and time of the diagnosis significant in multivariate analysis. In patients diagnosed between 1992 and 2009, fracture at diagnosis remained as a significant factor for OS in multivariate analysis. In patients diagnosed between 2010–2024, the given bone disease treatment was a significant factor for both TTLF and OS and for OS, also delay in starting of bone disease treatment was a significant factor.

**Table 2:** Unadjusted univariate and multivariate analysis of the factors impacting time to later fracture (TTLF) and overall survival (OS). HR Hazard Ratio. CI Confidence Interval.

	Univariate	Multivariate	HR	CI
**TTLF**				
Time of the diagnosis	<0.001	0.775	0.948	0.658–1.367
Bone disease at diagnosis	0.005	0.043	1.576	1.014–2.450
Fracture at diagnosis	0.006	0.128	1.284	0.930–1.772
Bone disease treatment	<0.001	0.023	1.243	1.030–1.498
**OS**				
Time of the diagnosis	0.031	<0.001	2.354	1.645–3.369
Bone disease treatment	0.003	0.134	1.142	0.960–1.360
Delay in starting of bone disease treatment	<0.001	0.004	0.543	0.359–0.822
Reason for the delay of bone disease treatment start	0.008	0.421	1.029	0.960–1.103

Diagnosis made between 1992 and 2009				
**TTLF**				
Bone disease at diagnosis	<0.001	0.100	2.394	0.847–6.767
Calcium plus vitamin D supplementation before diagnosis	0.010	0.171	1.551	0.827–2.908
Bone disease treatment	0.031	0.440	1.145	0.813–1.612
**OS**				
Bone disease at diagnosis	0.030	0.686	1.133	0.620–2.070
Fracture at diagnosis	0.024	0.012	1.767	1.135–2.750

Diagnosis made between 2010 and 2024				
**TTLF**				
Bone disease treatment	0.049	0.24	1.409	0.911–2.182
Delay in starting of bone disease treatment	0.016	0.124	1.461	1.052–2.029
**OS**				
Bone disease treatment	0.026	0.002	1.425	1.140–1.783
Delay in starting of bone disease treatment	<0.001	0.017	0.569	0.358–0.903
Reason for the delay of bone disease treatment start	0.017	0.564	1.024	0.946–1.108

Calcium plus D-vitamin supplement was started before MM diagnosis for 128 (20.5 %) patients and for 428 (68.5 %) patients at the time point of multiple myeloma diagnosis. A great majority of the patients received zoledronic acid (n = 363, 58.1 %), and 81 (13.0 %) patients received denosumab and 134 (21.4 %) other therapy to treat myeloma bone disease.

### 3.1 Later myeloma-related skeletal event

A total of 181 (29.0 %) patients developed a later fracture and of those 116 (64.1 %) were pathological, 30 (16.6 %) traumatic and 29 (16.0 %) osteoporotic fractures. For six (3.3 %) patients' information on fracture type was not available. Of the 460 patients that were diagnosed with myeloma between 2010 and 2024, 102 (22.2 %) experienced later fracture with a median of 27 months. Of those diagnosed between 1992 and 2010 incidence (47.9 %, n = 79) was higher with a median TTLF of 43 months (p < 0.001). From patients with no fracture at diagnosis (n = 315), altogether 75 (23.8 %) developed a later fracture and from the 282 patients that were diagnosed with fracture at diagnosis, a total of 102 (36.2 %) developed a later fracture. Information on later fracture was not available from 72 (22.8 %) patients from the first group and from 44 (15.6 %) patients from the second group. Four out of the total number of later fractures were diagnosed in patients without information on diagnosis state fractures.

### 3.2 Dental care

At diagnosis, 140 (22.4 %) patients had removable teeth, and 63 (10.1 %) patients were toothless. Overall, patients were promptly referred to a dentist within three days (range 0–374) after myeloma diagnosis. Counted from the referral date, the median time to orthopantomogram (OPTG) was six days (range 0–147) and to dental appointment seven days (range 0–147).

### 3.3 Delay in the start of bone disease treatment

The median delay to the start of the bone disease treatment from diagnosis was 48 days (range 1–5022), which was used as a cut-off of delay according to area under the curve (AUC) result (0.549 (CI 95 % 0.496–0.603, p = 0.072)). Reasons for the delay included tooth extraction (n = 106, 48.0 %), delay with unknown reason or other reason (n = 59, 26.7 %) and delayed access to a dentist (n = 7, 3.2 %). For 49 (22.2 %) patients' information on the reason for the delay was not available.

Among the 224 (35.8 %) patients without a delay, 69 (30.8 %) experienced a later fracture within a median time of 88 months (range 0–203) (p = 0.347). In contrast, of the 221 (35.4 %) patients with a delayed treatment start, 81 (36.7 %) had a later fracture and the median time to later fracture was 76 months (p = 0.347). Of those patients without a delay within two years, 18.8 % had experienced a fracture compared to 18.2 % in those with delay, and within five-years follow-up this rate rose to 33.9 % compared to 45.5 % (p = 0.347) in the patients in delay-group.

In addition, a total of 46 (7.4 %) patients developed osteonecrosis of the jaw (ONJ), of which 15 (32.6 %) were diagnosed in patients from the non-delay group and 27 (58.7 %) in patients from the delay group (p = 0.053). For four (8.7 %) patients with ONJ, more detailed information on dental care dates was missing and making it impossible to define the delay.

Furthermore, according to the delays in the treatment of bone disease, patients were divided into three groups: patients without a delay (n = 155), patients with a delay because of tooth extraction (n = 73) and patients with delay due to another reason (n = 73). ONJ was diagnosed in 15 (9.7 %) patients without a treatment delay, in 23 (31.5 %) patients with a tooth extraction as a cause for delay and in four (5.5 %) patients with a delay due to another reason (p < 0.001). Between these patient groups, there was no difference in later fracture incidence (p = 0.194). At a two-year mark, in the non-delay group 18.9 % had later fracture and at five-years after diagnosis the incidence was 34.2 %. For the patients who had a delay due to a tooth extraction, the corresponding numbers were 25.4 % and 46.1 % and for the patients experiencing a delay due to another reasons the numbers were 10.9 % and 44.6 % (p = 0.194), respectively. In patients with a tooth extraction as a reason for delay, the median time for bone disease treatment from diagnosis was three months (range 1–81).

For calcium and D vitamin supplement the median time to initiation after myeloma diagnosis was 9 days (range 1–4523). Diminished incidence of later fractures in patients who had prior treatment with calcium plus D vitamin did not reach statistical significance (p = 0.621). Furthermore, it had no impact on OS (p = 0.615). Within patients for whom calcium plus D vitamin was initiated within one month after myeloma diagnosis, there was a trend towards fewer later fractures when compared to those whom it was initiated later (194 (28.5 %) vs. 115 (43.5 %), p = 0.118).

### 3.4 Impact of bone disease related factors on OS

Imaging methods used to detect bone disease at diagnosis were compared between patients diagnosed in years 1992–2009 and those diagnosed between 2010–2024. Between 1992 and 2009, X-ray was used in 54.5 % of 165 patients, CT in 12.7 %, and MRI in 14.5 %, with imaging data unavailable for 18.2 % of patients. In contrast, for patients diagnosed between 2010 and 2024 (n = 460), X-ray was used in 15.2 %, CT in 49.1 %, and MRI in 23.9 %, with imaging data unavailable for 7.8 % of patients. The choice of imaging method had no impact on OS in patients diagnosed between 1992 and 2009 (p = 0.210). However, patients diagnosed between 2010 and 2024 who underwent MRI for bone disease detection experienced prolonged OS (p < 0.001).

**Fig. 2:**
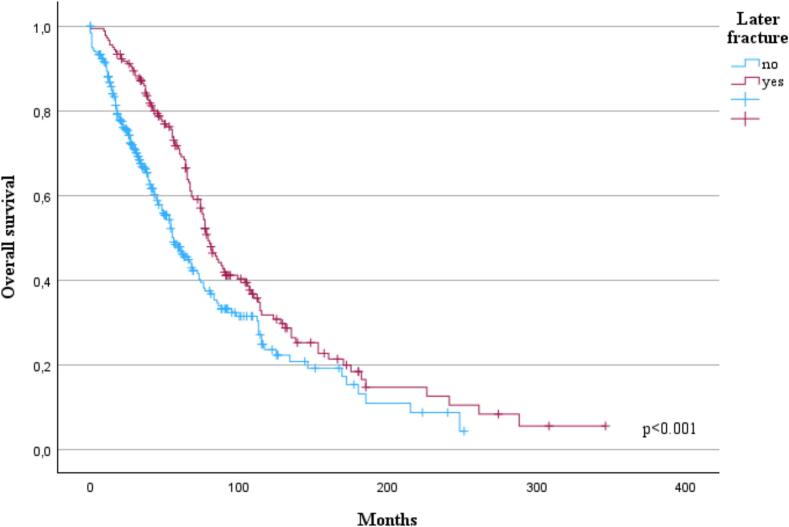
Effect of later fracture (n = 181) in myeloma patients on overall survival (OS). (p < 0.001.).

A presence of bone disease at diagnosis had no statistically significant impact on OS (p = 0.100) However, patients with a later fracture had prolonged OS (p < 0.001, Fig. 2) and this result remained when analyzed only patients diagnosed between 2010–2024 (p = 0.024) but not within patients diagnosed between 1992 and 2009 (p = 0.173).

**Fig. 3:**
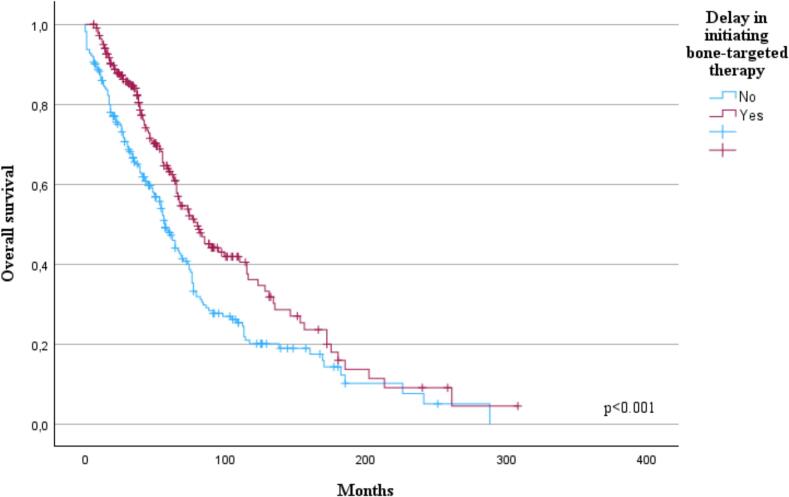
Impact of timing of bone-targeted therapy on overall survival (OS). Patients with a treatment delay (n = 221, 35.4 %) in initiating bone-targeted therapy experienced improved OS (p < 0.001).

Unexpectedly, patients with a delay in starting bone disease treatment experienced a better OS (p < 0.001, Fig. 3). This result persisted in patients diagnosed between 2010 and 2024 (p < 0.001) but not among patients diagnosed between 1992–2009 (p = 0.102). Different anti-myeloma treatments had no significant difference on OS (p = 0.056) within the whole study population. However, when comparing anti-myeloma treatments’ impact on OS within patients diagnosed between 2010 and 2024 those who received PI-based treatment seemed to have OS benefit (p < 0.001).

In the comparison of bone disease treatments and their impact on OS, patients treated with denosumab experienced significantly poorer OS compared with zoledronic acid and other bone disease treatments (p < 0.001). This result remained consistent in patients diagnosed with myeloma between 2010 and 2024 (p < 0.001).

### 3.5 Impact of initial bone disease on later fractures

**Fig. 4:**
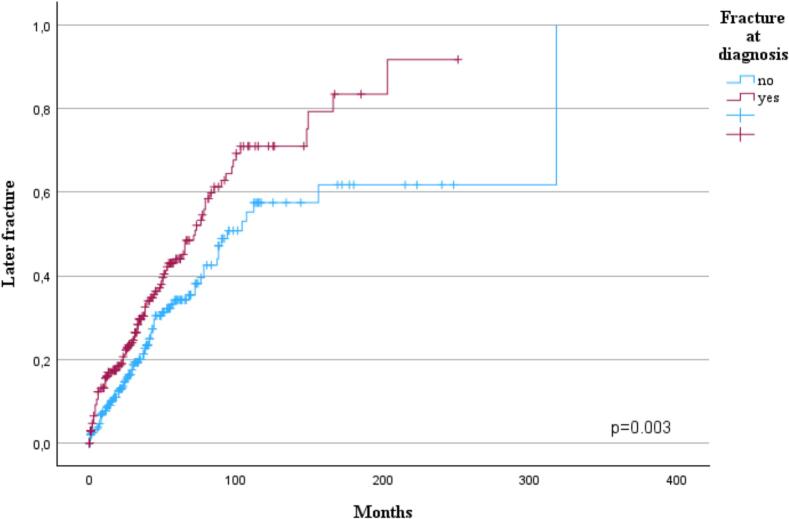
Influence of a fracture at diagnosis (n = 282) on later fractures.

**Fig. 5:**
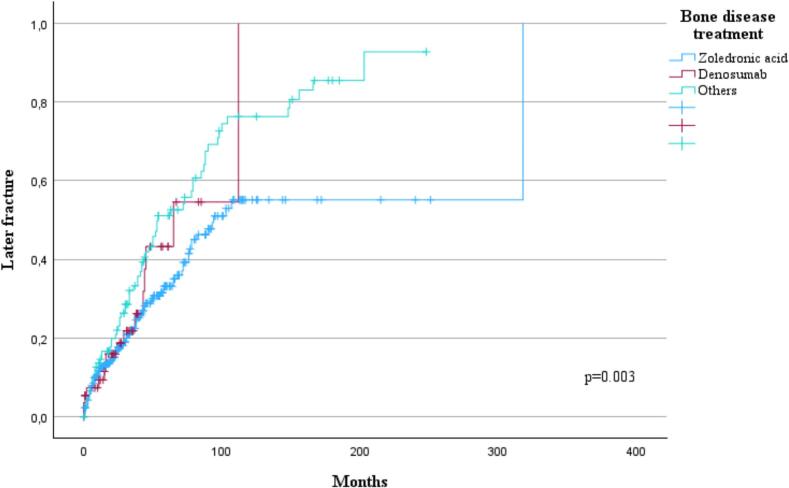
Time to later fracture according to myeloma bone disease targeted drugs used. Patients treated with zoledronic acid experienced longer time to later fracture (TTLF) (p = 0.003).

Patients with a fracture at diagnosis experienced later fractures earlier (p = 0.003, Fig. 4). This result was consistent in patients diagnosed between 1992 and 2009 (p = 0.009) but not with patients diagnosed between 2010–2024 (p = 0.193). However, the pathological nature of fracture at diagnosis had no impact on later fractures when compared to traumatic or osteoporotic fractures (p = 0.310). Fracture at diagnosis did not have impact on OS (p = 0.214) even if the fracture was pathological (p = 0.143). Patients with bone disease at diagnosis experienced later fractures more frequently and earlier than patients without a bone disease at diagnosis (p < 0.001). This result persisted with patients diagnosed between 1992 and 2009 (p < 0.001) but not in those diagnosed between 2010 and 2024 (p = 0.121). There was no statistical difference in the incidence of later fractures when patients with or without a fracture at the time of diagnosis were compared based on whether osteoprotective treatment was delayed or not (p = 0.455, p = 0.174). Patients treated with other drugs or denosumab experienced earlier later fractures than those treated with zoledronic acid (p = 0.003, Fig. 5). However, this difference was not observed when comparing those patients diagnosed between 1992–2009 (p = 0.208) and those diagnosed between 2010–2024 (p = 0.167).

## 4 Discussion

The aim of the current study was to examine the delays in treating multiple myeloma-related bone disease, identify the causes of these delays and evaluate their impact on patients’ long-term prognosis. The present study showed a median 48-day delay in the start of targeted bone disease drug and a main reason for the delay was tooth extraction. Moreover, fracture or bone disease at diagnosis predicted an earlier occurrence of later fractures. In addition, we found that patients with shorter delay in the start of their myeloma bone disease treatment had better prognosis.

The interaction between osteoclasts and osteoblasts to form bone tissue is mainly regulated by cytokines secreted by osteocytes [9]. Especially RANKL, a cytokine and humoral factor, is recognized as a central mediator for osteoclasts with increased action in MM [10]. It binds to its receptor RANK inducing bone degradation [11]. In MM, an enhanced specialization and function of osteoclasts and suppressed action of osteoblasts, lead to minimal bone formation and osteolytic lesions [12]. Furthermore, bone degradation and osteoclasts excrete growth factors that promote the growth of MM cells [1,10]. Extensive bone destruction results in hypercalcemia which is related to more aggressive MM disease and associates with poorer prognosis [13].

Bone disease is found in over 80 % of the NDMM patients at the timepoint of diagnosis and pathologic fractures will develop in up to 60 % of MM patients during the disease course [12,14]. In a comparative study of imaging methods, CT imaging detected 24 % more bone changes compared to traditional skeletal imaging [15]. Modern imaging methods such as MRI, CT and PET-CT provide complementary information in many patients [16]. In the present study, CT was used to detect bone disease in a great majority of patients. However, the use of x-ray imaging in earlier years may hamper the results due to the insufficient sensitivity to detect small bone lesions. Also, the tempo of imaging procedures in the follow-up according to updated recommendations may have an impact on results. This may have association with the fact that within patients diagnosed between 1992–2009 TTLF was longer than in patients diagnosed during 2010–2024 in the present study.

Bisphosphonates, such as zoledronic acid and pamidronate, prevent SREs by targeting areas of high bone resorption, inhibiting osteoclast differentiation and facilitating apoptosis [13,17] resulting in reduced bone pain, SREs and hypercalcemia. ZA binds to hydroxyapatite [18] in bone where osteoclast absorb it leading to their apoptosis. Denosumab acts as a RANKL inhibitor reducing bone resorption by inhibiting osteoclast genesis and activity [10]. Denosumab has shown equivalent efficacy to ZA in preventing SREs without difference in OS or PFS [19,20].

The IMWG guidelines recommend all patients with NDMM or relapsed or refractory myeloma to receive monthly zoledronic acid as a bone-specific treatment despite myeloma bone disease diagnosis [6] at least 12 months administration according to the response of MM treatment. Denosumab is recommended for patients with renal impairment but only if bone disease is diagnosed with imaging studies. Rebound effect must be kept in mind before denosumab discontinuation. Calcium and vitamin D supplementation is recommended for all patients receiving bisphosphonates or denosumab [6]. PFS may be protracted in patients with NDMM, myeloma-related bone disease and who are suitable for autologous hematological stem cell transplantation [21]. In the present study, patients treated with denosumab experienced significantly poorer OS. This may partly be explained by the fact that denosumab was often administered to patients with severe renal insufficiency, severe hypercalcemia or another condition deteriorating overall health. Furthermore, patients receiving denosumab or other bone disease treatment experienced earlier later fractures (p = 0.003).

In a recent population-based study patients with fracture at diagnosis had 28 % higher risk for death and a later fracture doubled the risk for death in MM patients [1]. The risk of death related to fracture in diagnosis was highlighted especially in patients under 70 years old at MM diagnosis. The same association was seen in our previous study, where the poorer OS was associated with diagnosis of bone disease and fracture [22]. In the present study, 29 % had a later fracture and contrary to the results reported in previous literature [1,23] these patients had prolonged survival. This result persisted in patients diagnosed between 2010 and 2024 but not in those diagnosed during 1992–2009. These results can be partially explained by the development of novel armamentarium, particularly antimyeloma treatment. However, the potential influence of selection bias and the small sample size should also be considered. Instead, bone disease at diagnosis had no impact on OS in our study.

A standard time for delay of the initiation of bone disease treatment is not defined in the international scientific literature. The median delay in the present study was 48 days and as expected, the major reason for delay in starting targeted bone disease therapy was tooth extraction. Instead, we found no delay regarding the dentist's appointment. One third of the study population with optimal dental health had delay for the start of the bone disease treatment for unknown reason. The importance of good dental care cannot be overemphasized for patients with myeloma and clinician should pay attention to timely initiation of targeted bone disease drugs.

A previous study revealed that, two years after diagnosis, the SRE rate was 74.6 % in patients who experienced >60 day delay in administration of zoledronic acid compared to 56.5 % in those who received early (<60 days) zoledronic acid treatment [8]. In the present study, at five years after MM diagnosis the proportion of patients who experienced later fracture was comparable (33.9 vs. 45.5 %) between the patients with timely and delayed start of bone disease therapy.

Osteonecrosis of the jaw (ONJ) is characterized by exposed necrotic bone causing pain, soft tissue failure to heal, infection and dental instability [23]. The risk can be reduced by avoiding invasive dental procedures during bone disease treatment monitoring dental status before and during bone disease treatment [24]. Risk of ONJ rises with prolonger bisphosphonate usage and likely with ZA [25]. A most recent retrospective study concluded in MM patients treated with chemotherapy and corticosteroids that invasive dental procedures are major risk for ONJ during BP treatment [7] and myeloma-derived bone disease in jawbones was linked with poorer prognosis [7]. In the present study, ONJ was diagnosed in 7.4 % and 26.0 % of those with a tooth removed in at diagnosis. A relatively larger number of ONJ were diagnosed in patients whose osteoprotective treatment had been delayed due to tooth removal. We do not have plausible explanation for the relatively large amount of ONJ patients, but possibly despite of delay, the start of bone disease treatment has realized before healing of the gum. A multicenter study reported an 8.8 % incidence of medication-related ONJ (MRONJ) among patients receiving antiresorptive treatment. The incidence was higher with denosumab (11.6 %) than bisphosphonates alone (2.8 %) [26]. In addition, a *meta*-analysis found 5 % incidence of MRONJ with bisphosphonates only and 4 % with denosumab alone [27]. Of note, in the present study, ONJ had no statistically significant impact on OS.

Some limitations of the present study must be acknowledged. The retrospective nature of the study should be considered when interpreting the results. For some of the patients, data concerning dental treatment was not available since treatment was provided by a private dentist or by the patient’s municipality. Also, exact information concerning some cause of deaths was missing. In addition, for some patients in previous years, X-ray was used to diagnose bone disease which, according to current knowledge, does not have sufficient sensitivity to all bone changes. However, between patients diagnosed in 1992–2009, there was no difference in OS depending on the imaging method used for bone disease detection. To enhance the reliability of our results further information on the dosage and the duration of bone-specific therapy received by patients should be collected. The strengths of the study include a real-world setting into the detailed treatment of MM patients, including the time of the referral to the dentist with the time periods from the diagnosis of the myeloma to specific time points before the start of the bone disease treatment and a long follow-up.

To conclude, this study provides large real-world data supporting that fracture or bone disease at diagnosis predict earlier later fractures. Patients with shorter delay in the start of their myeloma bone disease treatment seemed to have better prognosis. Considering dental health, further studies are needed to investigate the optimal time frame to start targeted bone disease treatment after myeloma diagnosis.

## Data availability statement

The original data is available by reasonable request.

## Funding

This work was supported by Finnish Hematology Society (VE).

## Declaration of competing interest

The authors declare the following financial interests/personal relationships which may be considered as potential competing interests: Veera Eskelinen reports financial support was provided by Finnish Society of Hematology. Milla Kuusisto reports financial support was provided by Relander Foundation sr. Milla Kuusisto reports financial support was provided by The Finnish Medical Foundation. Milla Kuusisto reports financial support was provided by Thelma Mäkikyrö Fund. Anu Partanen reports a relationship with Behring that includes: consulting or advisory and speaking and lecture fees. Anu Partanen reports a relationship with Abbvie that includes: board membership, consulting or advisory, and speaking and lecture fees. Anu Partanen reports a relationship with Janssen-Cilag that includes: funding grants and travel reimbursement. Anu Partanen reports a relationship with Novartis that includes: funding grants and travel reimbursement. Anu Partanen reports a relationship with Pfizer that includes: funding grants and travel reimbursement. Anu Partanen reports a relationship with Takeda that includes: funding grants and travel reimbursement. If there are other authors, they declare that they have no known competing financial interests or personal relationships that could have appeared to influence the work reported in this paper.
